# Surface Defect Detection Based on Adaptive Multi-Scale Feature Fusion

**DOI:** 10.3390/s25061720

**Published:** 2025-03-10

**Authors:** Guochen Wen, Li Cheng, Haiwen Yuan, Xuan Li

**Affiliations:** School of Electrical and Information Engineering, Wuhan Institute of Technology, Wuhan 430205, China; 22303010042@stu.wit.edu.cn (G.W.); hw_yuan@wit.edu.cn (H.Y.); lixuan@wit.edu.cn (X.L.)

**Keywords:** adaptive multi-scale feature fusion (AMSFF), preprocessing, surface defect detection, salient object detection

## Abstract

Surface defect detection plays a quality assurance role in industrial manufacturing processes. However, the diversity of defects and the presence of complex backgrounds bring significant challenges to salient object detection. To this end, this study proposes a new adaptive multi-scale feature fusion network (AMSFF-Net) to solve the SOD problem of object surface defects. The upsampling fusion module used adaptive weight fusion, global feature adaptive fusion, and differential feature adaptive fusion to fuse information of different scales and levels. In addition, the spatial attention (SA) mechanism was introduced to enhance the effective fusion of multi-feature maps. Preprocessing techniques such as aspect ratio adjustment and random rotation were used. Aspect ratio adjustment helps to identify and locate defects of different shapes and sizes, and random rotation enhances the ability of the model to detect defects at different angles. The negative samples and non-uniform-distribution samples in the magnetic tile defect dataset were further removed to ensure data quality. This study conducted comprehensive experiments, demonstrating that AMSFF-Net outperforms existing state-of-the-art technologies. The proposed method achieved an S-measure of 0.9038 and an $F_{\beta }^{max}$ of 0.8782, which represents a 1% improvement in $F_{\beta }^{max}$ compared to the best existing methods.

## 1. Introduction

In recent years, computer vision technology has made significant progress, which provides strong technical support for the field of salient object detection. Existing image processing techniques realize automatic detection and classification of defects such as blowhole, break, crack, and fray through the analysis of object surface images, which has become a research hotspot.

Traditional image processing techniques typically rely on edge detection, texture analysis, and morphological operations [1,2,3,4]. While effective in specific scenarios, these methods are limited in robustness and generalization, making it challenging to handle complex and variable real-world production environments. The emergence of deep learning technology [5], particularly the widespread adoption of Convolutional Neural Networks (CNNs) [6], has shifted the focus of defect detection methods toward deep learning [7].

In recent years, researchers have proposed object detection models such as Faster R-CNN [8], YOLO [9], and SSD [10]. These models have shown promising results in both public datasets and actual production environments. However, Faster R-CNN may overlook small defects during candidate region generation, resulting in suboptimal performance for detecting small-sized defects. When dealing with multi-scale defects, YOLO may struggle to effectively detect both large and small defects simultaneously. Additionally, SSD is susceptible to false detections under complex backgrounds, especially when object surfaces exhibit interference factors like texture variations or changes in illumination, leading to poor model robustness.

Lu et al. [11] proposed Resformer-Unet, a U-shaped encoder–decoder framework that integrates CNN and Transformer to concurrently extract multi-scale features, enhancing the capture of global and local information. However, this method still faces certain limitations when detecting significant defects. For example, the feature fusion module in their approach primarily relies on simple operations like feature stacking and concatenation, without considering the importance weighting of different features. Additionally, there is a lack of an adaptive mechanism to adjust the feature fusion strategy based on the specific characteristics of the image. Furthermore, although the framework uses CNN and Transformer in parallel, the fusion of features from the two branches is overly mechanical, lacking a mechanism to assess the quality or contribution of features at different scales. Tang et al. [12] investigated a steel plate surface defect detection method based on the Swin Transformer and Feature Pyramid Network (FPN), which generates Regions of Interest (ROIs) for defects by fusing multi-scale feature maps. However, the method in that paper fixes all features to a dimension of 256, ignoring the inherent characteristics of features at different scales. In addition, the simple interpolation-based upsampling process in that paper fails to fully capture the semantic information of the features. Furthermore, the method lacks a cross-scale feature enhancement mechanism and attention mechanisms to highlight important features, such as spatial attention for emphasizing key areas and adaptive feature selection. These issues limit the method’s performance, especially in complex background scenarios. Therefore, further improvements are needed to better utilize multi-scale information and enhance accuracy under such challenging conditions. Zhao et al.’s [13] Feature-Aware Network (FaNet) achieves few-sample defect classification through the feature-attention convolution module (FAC) and the online feature enhancement integration module (FEI). However, in the model design, the feature fusion method is overly static, not considering the importance of input features or dynamically adjusting the fusion strategy based on the contribution of features at different layers and scales. Moreover, the model does not explicitly consider the interaction between features at different scales. As the network deepens, the spatial resolution of features gradually decreases, and the model becomes more reliant on the global information from higher-level features, neglecting the flow of information across scales. This can lead to information loss or the insufficient utilization of lower-level features, particularly when dealing with complex backgrounds or fine-grained features.

In the field of deep learning, various network architectures are available for use. Among them, ResNet [14], as a classic network architecture, effectively alleviates common issues of gradient vanishing [15] or explosion during the training of deep networks by introducing residual connections. However, despite ResNet’s excellent performance in many tasks, its network structure is relatively complex and heavily dependent on deep-layer features. This may lead to the neglect of fine-grained local features in defect detection, which is crucial for the precise detection of micro-defects on the magnetic tile surface in this paper.

In recent years, the Transformer architecture has made significant progress in computer vision tasks due to its exceptional global modeling capabilities [16]. Transformer is particularly adept at capturing long-range dependencies in images. However, its lower computational efficiency and the need for large-scale training data can result in high computational costs and resource consumption in practical applications, especially in tasks involving high-resolution images or small datasets. As such, the Transformer architecture is not the optimal choice for the task of magnetic tile surface defect detection.

In contrast, VGG16, with its simple and efficient layer-by-layer convolutional structure, is highly suitable for the detection of magnetic tile surface defects due to its characteristic of progressively expanding the receptive field. Through successive convolution and pooling operations, VGG16 effectively extracts multi-scale local features, which is critical for recognizing minute defects. Additionally, the linear forward propagation path of VGG16 maintains the continuity of local features, which is advantageous for capturing subtle texture features. Compared to deeper networks such as ResNet, VGG16 is simpler in structure, offers better training stability, and allows for faster and more efficient feature extraction with fewer computational resources.

Therefore, considering the high dependence on local details in magnetic tile surface defect detection and the characteristics of the limited dataset, this paper selects VGG16 as the backbone network for feature extraction. The goal is to improve the detection accuracy of micro-defects through its efficient local feature extraction capabilities.

This paper addresses the issue of improving the detection accuracy of micro-defects on limited datasets by proposing an adaptive multi-scale feature fusion network (AMSFF-Net). AMSFF-Net consists of three components: adaptive weight fusion, global feature adaptation fusion, and differential feature adaptation fusion. The adaptive weight fusion component dynamically adjusts weights based on the significance of features to enhance the effectiveness of feature fusion. The global feature adaptation fusion component dynamically adjusts feature weights according to their importance, improving the effect of feature fusion. Finally, the differential feature adaptation fusion mechanism effectively captures variations among different features, enhancing their expressive capability.

## 2. Data Preprocessing and Filtering

Preprocessing plays a key role in salient object detection. The accuracy of saliency detection directly depends on data quality and feature validity. Considering that defects of various shapes and sizes can appear on object surfaces, as well as other interfering factors such as blotching or illumination changes, accurate preprocessing is essential to ensure that the model can accurately identify and locate defect regions. Effective preprocessing helps to remove noise, balance the data, enhance features, and improve the robustness and generalization of the model.

### 2.1. Dataset Filtered

**Figure 1:**
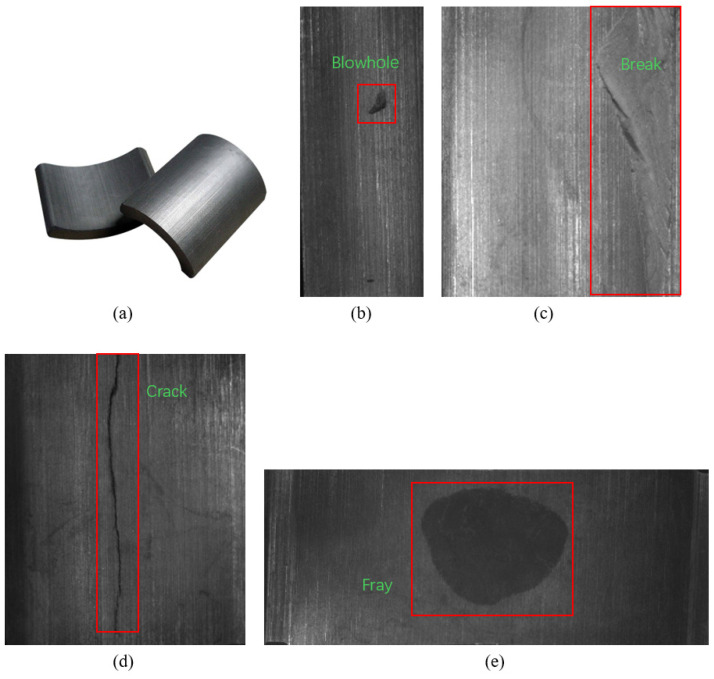
Magnet tile image and images of four types of magnet tile defects: (**a**) normal magnetic tiles, (**b**) blowhole defect: Subsurface or surface cavities formed by gas entrapment during the casting process, (**c**) break defect: Complete through-thickness fracture in magnet tiles, typically characterized by penetrating discontinuity, (**d**) crack defect: Linear fissures manifesting on or beneath the material surface, and (**e**) fray defect: Surface delamination or fibrous surface morphology observed in magnet tiles.

Since this paper aims to improve the detection accuracy of subtle defects using small datasets, the Magnetic-Tile-Defect dataset was chosen as it provides a suitable foundation for investigating these challenges. The dataset includes images of six types of defects: blowhole, break, crack, fray, free, and uneven. For our study, we selected four types of defects, namely, blowhole, break, crack, and fray, resulting in a total of 289 images. These four types of defect images, along with the normal magnetic tile images, are shown in Figure 1. These defects are particularly relevant to the context of detecting minor imperfections in tile surfaces, which is the focus of this research.

In this paper, we used the publicly available Magnetic-Tile-Defect dataset [17] as the base dataset, and through processing this dataset, we created a refined dataset for model validation. To ensure effective model training and research focus, this paper excluded negative and uneven samples from the Magnetic-Tile-Defect dataset, which includes defect images of blowholes, breaks, cracks, and frays on tile surfaces after the exclusion of these samples. Eliminating negative samples addresses the issue of class imbalance commonly encountered in image processing, which may lead to the model overfitting to the majority class and diminishing its ability to recognize minority class instances—the defect samples emphasized in this paper. Additionally, this study concentrated on identifying and locating specific types of defects on object surfaces; thus, uneven samples, which may arise due to normal variations during production rather than being indicative of material or manufacturing process flaws, were excluded. Their inclusion could introduce irrelevant noise, interfering with the accurate learning and identification of true defects by the model. Therefore, for enhanced accuracy and generalization capability, we renamed the refined dataset as Focused-Defect-Detection-Tiles.

### 2.2. Dataset Preprocessing

In this study, the original dataset underwent random rotation and aspect ratio adjustment. The introduction of random rotation is based on the fact that object surface defects can occur at any angle in actual production environments. By randomly rotating the images within the range of [−180°, 180°], data diversity is increased, enabling the model to better adapt to defects at various angles.

We introduced improved joint image and mask adjustment methods to effectively handle defects of different shapes and sizes. Specifically, we designed a class called JointResize, which simultaneously adjusts both the image and mask to a specified size while providing an option to maintain the aspect ratio of the original image. This approach allows scaling without distortion, preserving geometric defect features more accurately.

When maintaining the aspect ratio, we first calculated the scaling ratio so that either the long or short side of the image matches the target size, followed by the proportional scaling of both dimensions equally. If, after scaling, there was a mismatch between image size and target size, blank areas were filled around the image to ensure that the final images have the desired dimensions. This method not only increases dataset diversity but also effectively prevents overfitting by exposing models during training to more deformed and rotated defect samples, thus improving their robustness and generalization ability in practical applications. With this improved preprocessing step, the performance of detecting object surface defects significantly improved, laying a solid foundation for subsequent experiments and model evaluation.

## 3. Proposed Methodology

### 3.1. Overall Architectural Design

**Figure 2:**
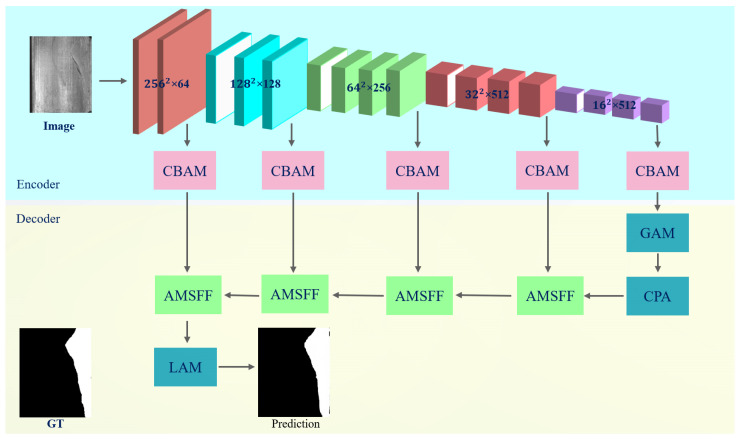
The architecture diagram of AMSFF-Net. In this study, VGG-16 is used as a feature extractor to generate feature maps at five different levels, which are processed by the CBAM attention mechanism. AMSFF represents the adaptive multi-scale feature fusion module, GAM denotes the Global Attention Module, CPA module is a Cascaded Pyramid Attention module, and LAM represents the Local Attention Module.

The VGG16 [18] network, depicted in Figure 2, was chosen as the backbone network for salient object detection in this study due to its exceptional feature extraction capability. With a simple structure and outstanding performance, the VGG16 network has been widely employed in image processing. In this study, five feature maps at different levels were extracted from the VGG16 network denoted as $f_{r}^{i}$, where i ∈ {1, 2, 3, 4, 5}. These feature maps progressively enhanced their semantic richness from shallow to deep layers, providing abundant information for subsequent defect detection.

This study introduced the Convolutional Block Attention Module (CBAM) [19] attention mechanism to further enhance the model’s ability to focus on crucial features. The CBAM sequentially reweights the feature map through spatial [20] and channel attention [21] mechanisms, effectively enhancing sensitivity towards defect areas. Incorporating this attention mechanism enabled the network to concentrate more on key regions of an image, thereby improving defect detection accuracy.

After performing feature extraction and attention weighting, this study placed particular emphasis on the deepest feature map $f_{r}^{5}$ due to its inclusion of high-level semantic information, which is crucial for identifying intricate defect patterns. Subsequently, $f_{r}^{5}$ underwent further processing including multi-scale feature fusion and global autocorrelation processing to enhance the model’s ability in capturing global information and predicting context accurately.

Ultimately, an adaptive multi-scale feature fusion strategy was employed in order to fully utilize the diverse levels of feature maps by gradually integrating deep and shallow features. Through this hierarchical fusion strategy, the proposed model could effectively detect various surface defects of objects.

### 3.2. Adaptive Upsampling Fusion Module

The core component of AMSFF-Net is the adaptive weight fusion mechanism, which is implemented through the following steps: Firstly, convolutional layers are employed to individually convolve the left and right input feature maps for obtaining corresponding feature representations at different levels. This step aims to extract diverse features that provide rich representations for subsequent fusion processes. Subsequently, a simple addition operation is performed on the left and right feature maps to generate a combined feature map that captures complementary information between both views. The specific expressions are represented by Equations (1) and (2):

(1) $$X_{feat}=Conv(X)$$

(2) $$C_{feat}=L_{feat}+R_{feat}$$

**Figure 3:**
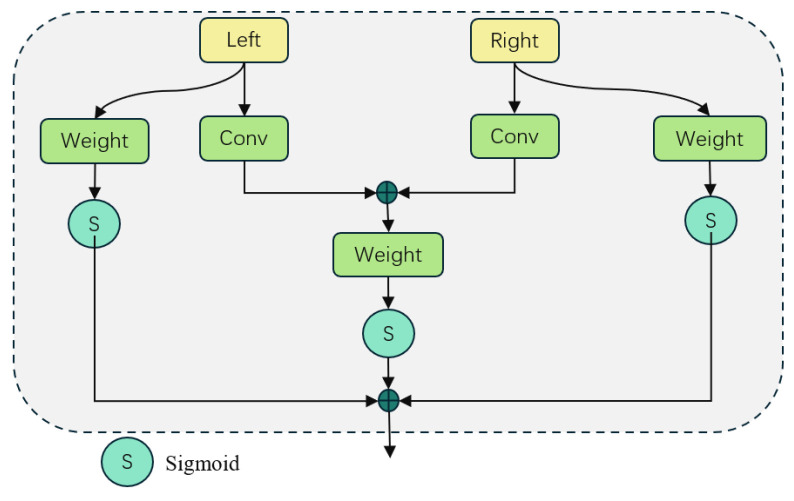
The computation of weight coefficients.

As shown in Figure 3, for each feature map (left, right, and combined), we calculated their respective weights and then summed them to obtain the total weight. This process employs the Sigmoid activation function [22] because it smoothly limits the output weights, making the weight distribution more reasonable and avoiding the interference of extreme values.

**Figure 4:**
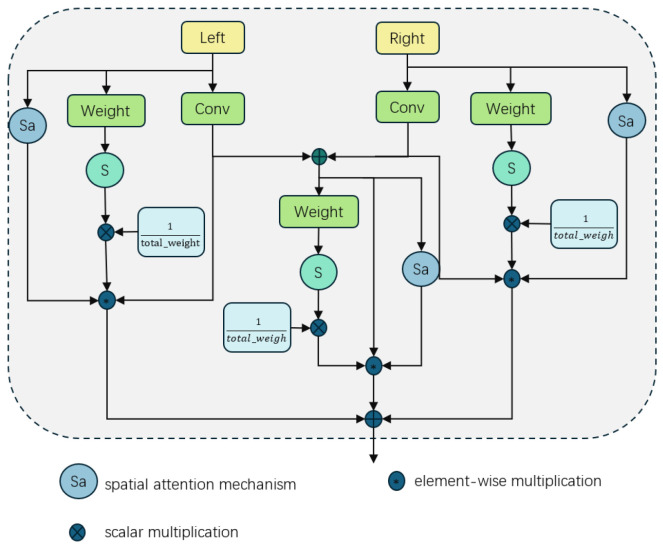
The technique of adaptive weight fusion.

As shown in Figure 4, the attention weight for each feature map was computed using the spatial attention mechanism (SA). SA effectively enables the model to focus on specific spatial regions within input data, facilitating enhanced extraction and fusion of crucial information while suppressing irrelevant or insignificant portions. Expressions such as (3) and (4) encapsulate these concepts. Finally, the adaptive weights derived from Equation (5) were integrated with attention weights to implement a weighted fusion of feature maps, as detailed in Equations (6) and (7). Specifically, the weight of each feature map was first normalized. This step guarantees the stability and efficiency of the fusion process. Subsequently, the normalized weights were multiplied by their corresponding feature maps and spatial attention weights to obtain weighted feature maps. All these weighted feature maps were then summed up to form the final output feature map. This adaptive weight fusion strategy empowers the network with automatic adjustment capabilities for fusion weights based on different feature maps’ importance levels, thereby enabling more accurate capture and integration of key information.

(3) $$W_{X}=\sigma (ConvWeight(X_{feat}))$$

(4) $$a_{X}=SA(X_{feat})$$

(5) $${\hat{W}}_{x}=\frac{W_{x}}{W_{total}}$$

(6) $$O_{x}={\hat{W}}_{x}\cdot X_{feat}\cdot a_{X}$$

(7) $$O=O_{L}+O_{R}+O_{C_{feat}}$$

The total weight, $W_{total}$, is calculated as the sum of individual weights $W_{L}$, $W_{R}$, and $W_{combined}$. The Sigmoid function σ is applied to obtain the final result. $\omega_{i}$ represents the computed weights.

**Figure 5:**
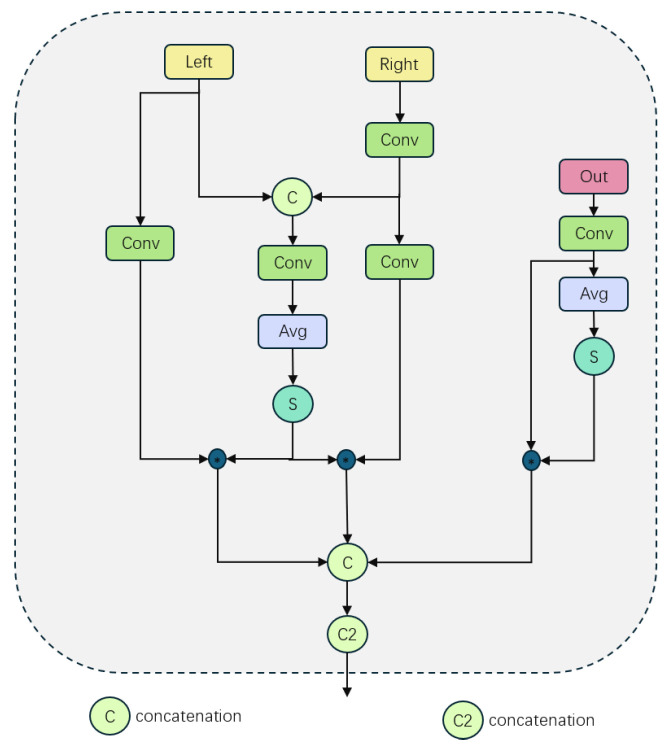
Enhanced integration of global features through adaptive fusion.

In the global feature adaptive fusion stage of AMSFF-Net, the feature fusion process was further enhanced. The primary objective of this stage was to calculate adaptive weights for global features, as illustrated in Figure 5, through global average pooling and Sigmoid function processing. This enabled more refined and effective feature fusion.

Firstly, the left channel feature *L* and right channel feature *R* were concatenated along the channel dimension to obtain *Concat*(*L*,*R*). Subsequently, this concatenated result was input into the convolutional layer *Conv_cat* for processing, resulting in a new feature map out1. This operation aimed at fusing features from both channels to fully leverage this information during subsequent processing steps. The expression can be represented by Equation (8).

(8) $$out1=Conv\_cat(Concat(L,R_{feat}))$$

In this case, $R_{feat}=Conv(R)$, where $R_{feat}$ represents the feature map obtained by applying a convolution operation *Conv* to *R*.

The fused feature map underwent a global average pooling operation, which aided in extracting an overall feature representation. The output of this pooling operation was then processed through a convolutional layer with a Sigmoid activation function. This layer played a crucial role in computing adaptive weights for each feature map (expressed as Equation (9)). By utilizing the Sigmoid function, we ensured that these weights fell within the range of 0 to 1, thereby achieving smoother weight distribution and mitigating any negative effects caused by mutations in weight on model performance.

(9) $$out_{gX}=\sigma (AvgPool(Conv(X_{input})))$$

$X_{input}$ can be either *out1* or *out*, and *AvgPool* represents the global average pooling layer.

The next step involved the element-wise multiplication of the calculated adaptive weight with the corresponding feature map, referred to as the weighted update of the feature map (expressed in Equation (10)). This process ensured that each feature map was adjusted according to its respective global feature weight, thereby enhancing important features and suppressing less significant ones. By incorporating this adaptive adjustment mechanism based on global feature weight, the model could accurately capture contextual information at a global level, leading to improved performance in terms of both feature fusion and overall model effectiveness.

(10) $$X_{updated}=Conv(X)\cdot out\_gX$$

The variable *X* can take on the values of *L*, *R*, or *out*, and the weighting coefficient *out_gX* corresponds to each value.

Finally, the updated feature maps were merged again to form the ultimate output feature map, as depicted in Equation (11). This fusion process was executed through a convolutional layer and Concat operation, ensuring the effective integration of information across diverse feature maps. By employing this multi-level and multi-strategy adaptive feature fusion approach, AMSFF-Net significantly enhanced the model’s capacity to comprehend and represent intricate scenes while maintaining computational efficiency.

$$out=Conv_{cat_{2}}•$$

(11) $$\left(Concat(L_{updated},R_{updated},{out}_{updated})\right)$$

**Figure 6:**
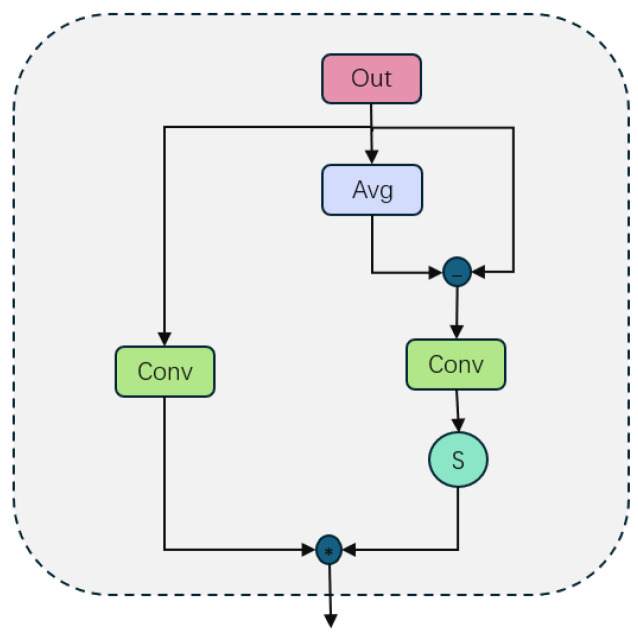
The adaptive integration of distinct features.

The differential feature adaptive fusion technique focuses on capturing the disparities among feature maps. By computing the discrepancy between the fused feature map and its global average pooling, followed by applying the Sigmoid function, we obtained an adaptive weight for differential features, as depicted in Figure 6. The expression for calculating the differential feature is given by Equation (12).

(12) out_g2=σ(Conv(out−AvgPool(out)))

After obtaining the adaptive weights of the differential features, we updated the feature maps by weighted element-wise multiplication, as described in Equation (13). This effectively enhanced the model’s focus on key local features.

(13) out=Conv(out)∗out_g2

The ASFF module effectively integrates features of different levels and resolutions through the above three adaptive fusion strategies, enhancing the network’s performance in complex visual tasks.

## 4. Experiment

(1) This study validated the effectiveness of the proposed method using the Focused-Defect-Detection-Tiles dataset, conducting comprehensive experiments and evaluations. This dataset was specifically designed for detecting defects on tile surfaces and encompasses various common types such as blowhole, break, crack, and fray that are frequently encountered in real-world industrial production.

(2) Experimental Setup: The hardware configuration for this study included an NVIDIA 4070 GPU with 8 GB of memory (Santa Clara, CA, USA), utilizing the PyTorch 1.13.0 framework [23]. During model training, each tile defect image was resized to 256 × 256 with three channels. Data augmentation techniques such as random horizontal flipping and random rotation were employed to mitigate the risk of overfitting. These augmentations also expanded the training dataset, providing a more diverse set of samples. Momentum Stochastic Gradient Descent (SGD) [24] was used to ensure the stability and convergence of the training process. The initial learning rate was set to 0.0015, combined with a polynomial decay strategy with a decay factor of 0.9, which helped gradually decrease the learning rate during training to achieve optimized results. The entire training process comprised 150 epochs to ensure the sufficient learning and optimization of the model parameters. Based on experimental considerations and hardware resources, the batch size was set to 8 to balance training efficiency and model performance. These settings allowed the network model to achieve good training results while maintaining efficiency. The specific neural network parameter settings are shown in Table 1.

Evaluation Metrics: Five different evaluation metrics were used in this study to comprehensively compare our proposed method with other existing saliency object detection (SOD) methods. These metrics encompassed a wide range of evaluation criteria, from structural similarity to error rate, ensuring a comprehensive assessment. The metrics used were S-measure ($S_{\alpha }$, α = 0.5) [25], Mean Absolute Error (MAE) [26], E-measure ($E_{\xi }$) [27], F-measure ($F_{\beta }$) [28], and weighted F-measure ($F_{\beta }^{w}$) [29]. Among them, the E-measure index is divided into average, adaptive, and maximum E-measure, which are denoted as mean $E_{\xi }$, adp $E_{\xi }$, and max $E_{\xi }$ respectively. The F-measure index is also divided into average, adaptive, and maximum F-measure, which are denoted as mean $F_{\beta }$, adp $F_{\beta }$, and max $F_{\beta }$, respectively.

S-measure ($S_{\alpha }$, α = 0.5): This metric assesses the structural similarity between the model output and the reference image, as shown in Equation (14).

(14) $$S_{\alpha }=\alpha *\;S_{o}+\left(1-\alpha \right)*\;S_{r}$$

In the S-measure formula, So represents the fundamental metric of structural information in the image, while Sr represents the fundamental metric of the image’s contrast information.

MAE: This is an accuracy metric for model evaluation, focusing on pixel-level predictions, as shown in Equation (15).

(15) $$MAE=\frac{1}{n}\sum_{i=1}^{n}\left|{\hat{y}}_{i}-y_{i}\right|$$

Here, n denotes the total number of samples, ${\hat{y}}_{i}$ is the predicted value from the model, and $y_{i}$ is the corresponding true value.

$E_{\xi }$: This metric evaluates both global and local performance of the model by integrating broad statistics with detailed pixel information, as shown in Equation (16).

(16) $$E_{\xi }=\frac{1}{\omega \times h}\sum_{x=1}^{\omega }\sum_{y=1}^{h}\varphi_{FM}(x,y)$$

In this formulation, h and ω denote the height and width of the map, respectively, where $\varphi_{FM}$ represents the enhanced alignment matrix.

$F_{\beta }$: This metric provides an assessment of the quality of saliency maps, as shown in Equation (17).

(17) $$F_{\beta }=\frac{\left(1+\beta^{2}\right)precision\;\cdot recall}{\beta^{2}\;\cdot precision+recall}$$

where $recall=\frac{TP}{TP+FN}$ and $precision=\frac{TP}{TP+FP}$.

$F_{\beta }^{w}$: This is an improved version of the traditional F-measure, which addresses some shortcomings of MAE and F-measure, offering more accurate evaluation results. In this experiment, $\beta^{2}$ was set to 1.0, following the recommendations in the literature [30], as shown in Equation (18).

(18) $$F_{\beta }=\frac{\left(1+\beta^{2}\right){precision}^{\omega }\;\cdot {recall}^{\omega }}{\beta^{2}\;\cdot {precision}^{\omega }+{recall}^{\omega }}$$

Using the above five evaluation indicators, the performance of AMSFF-Net in salient object detection could be comprehensively evaluated and compared, thus ensuring the effectiveness and practicability of the model.

**Figure 7:**
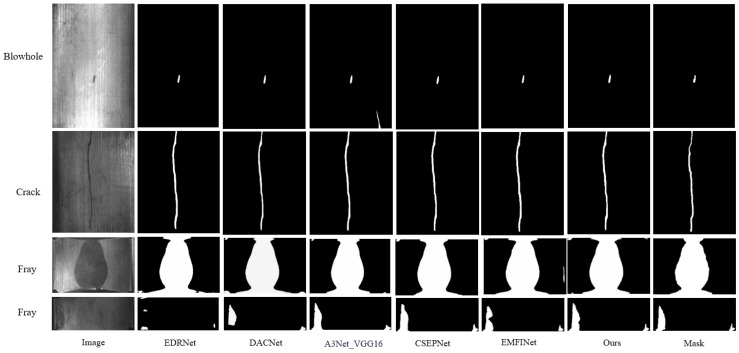
The saliency maps generated by the proposed method were compared with those produced by three other advanced methods.

**Figure 8:**
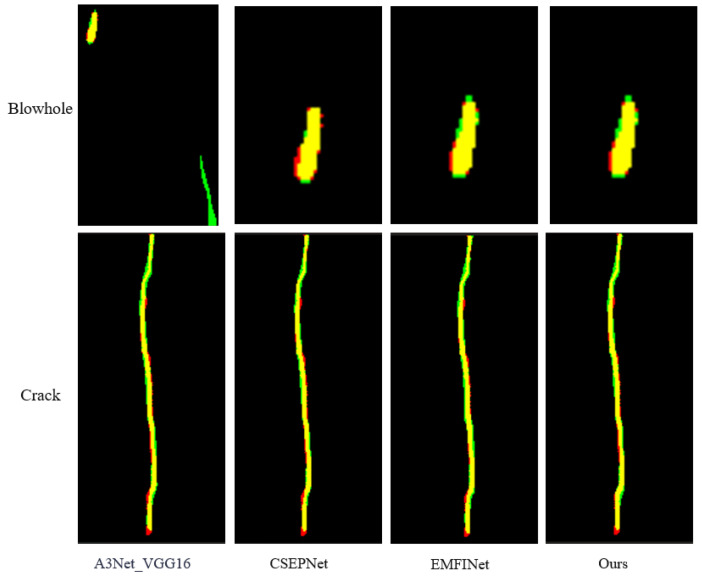
Comparison of the saliency maps generated by the proposed method and three other advanced methods for the blowhole and crack defect details.

**Figure 9:**
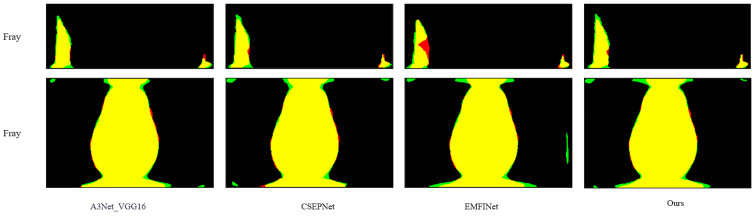
Comparison of the saliency maps generated by the proposed method and three other advanced methods for the fray defect details.

(3) This paper compared the proposed method with existing advanced SDI-SOD methods, including EDRNet [31], DACNet [32], EMFINet [33], CSEPNet [34], and two different architectures of A3Net [17] (A3Net_VGG16 and A3Net_Res2Net50). To ensure a fair comparison, all saliency maps were generated using open-source code with standard parameter configurations. A meticulous comparison procedure was designed in this study to ensure the reliability and validity of the evaluation results. Figure 7 shows a comparison of saliency maps generated by AMSFF-Net and other advanced methods. Additionally, Figure 8 and Figure 9 provide a detailed comparison between our method and the top three advanced methods. We overlay the saliency maps generated by these four methods with the ground truth images for comparison. The defects in the ground truth are highlighted in red, the generated saliency maps are shown in green, and the overlapping regions are displayed in yellow. The red and green portions in Figure 8 and Figure 9 represent the differences between the two overlapping images. From Figure 8, it can be observed that the performance of our method, based on the A3Net_VGG16 network, is comparable to that of the EMFINet and CSEPNet networks. However, as shown in Figure 9, our method outperforms both EMFINet and CSEPNet.

(4) Empirical Evaluation: Table 2 shows the experimental results of AMSFF-Net compared with other advanced methods. This paper employs nine metrics, namely, $S_{\alpha }$, M, $E_{\xi }^{adp}$, $E_{\xi }^{mean}$, $E_{\xi }^{max}$, $F_{\beta }^{adp}$, $F_{\beta }^{mean}$, $F_{\beta }^{max}$, and $F_{\beta }^{w}$. When evaluated on the Focused-Defect-Detection-Tiles dataset against eight representative methods, our proposed method slightly underperforms PANet on $E_{\xi }^{max}$, but outperforms the other methods across all other metrics. Furthermore, the performance of AMSFF-Net is significantly improved compared to the best performing EMFINet in the experiments. Specifically, AMSFF-Net achieves a 0.31% improvement in the $S_{\alpha }$ metric, indicating its slight superiority over EMFINet in detecting defect structural integrity, while maintaining the same MAE value, suggesting comparable pixel-level performance. In terms of $E_{\xi }$ metrics, AMSFF-Net shows improvements of 0.39%, 0.42%, and 0.5% in $E_{\xi }^{adp}$, $E_{\xi }^{mean}$, and $E_{\xi }^{max}$, respectively, demonstrating its enhanced capability in integrating broad statistical information with detailed pixel-level features, thus improving both global and local performance aspects. For $F_{\beta }$ metrics, AMSFF-Net exhibits notable advantages with improvements of 0.2% in $F_{\beta }^{adp}$, 0.5% in $F_{\beta }^{mean}$, and a substantial 0.94% in $F_{\beta }^{max}$, indicating enhanced precision and recall in defect identification. Additionally, the 0.75% improvement in $F_{\beta }^{w}$ further validates the model’s enhanced capability in balancing weighted precision and recall.

A comprehensive comparison between AMSFF-Net and EMFINet reveals several key advantages of the proposed method. While EMFINet demonstrates strong performance across a range of network architectures for defect detection tasks, the architectural improvements in AMSFF-Net lead to consistent gains across all evaluation metrics. The most notable improvement is observed in the $F_{\beta }^{max}$ metric, where AMSFF-Net achieves a 0.94% performance boost, indicating its ability to maximize both precision and recall simultaneously. This improvement is particularly significant, as $F_{\beta }^{max}$ is regarded as a key metric for evaluating a model’s ability to maintain high detection accuracy while minimizing false positives. Compared to EMFINet, AMSFF-Net shows a 0.39% to 0.5% improvement in the $E_{\xi }$ metrics, highlighting its superior capability in capturing both global context and local details, which is crucial for accurately delineating defect boundaries. Moreover, with the MAE remaining unchanged, the 0.31% improvement in the $S_{\alpha }$ metric suggests that AMSFF-Net achieves better structural prediction without sacrificing pixel-level accuracy. These improvements are attributed to the model’s enhanced feature fusion mechanism and adaptive multi-scale architecture, which enable AMSFF-Net to more effectively address the challenges in industrial defect detection when compared to EMFINet.

**Figure 10:**
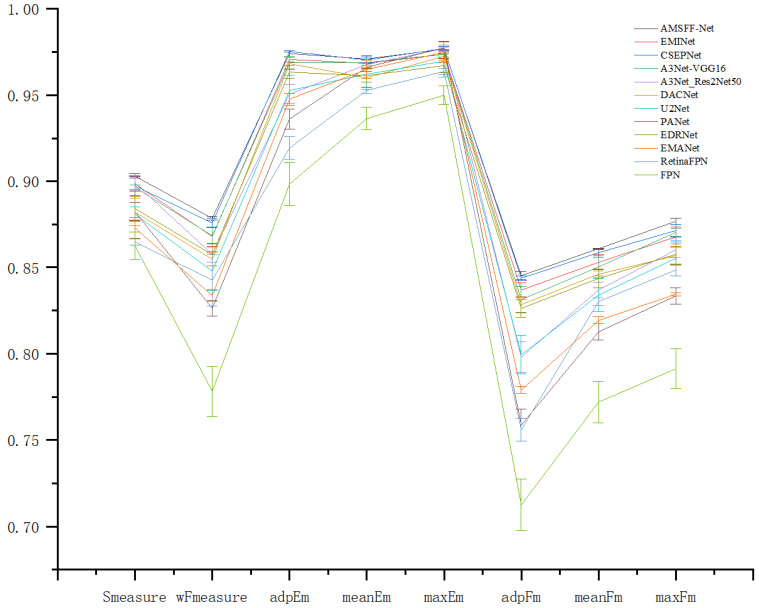
Error bar chart comparing AMSFF-Net with 11 other advanced methods.

(5) Error Analysis: Figure 10 presents the error bar chart comparing our proposed method with 11 advanced methods. As depicted in the figure, our method significantly outperforms several other state-of-the-art approaches in the $F_{\beta }^{max}$ metric, while showing slight superiority over other leading methods in metrics such as $S_{\alpha }$, $F_{\beta }^{w}$, and $E_{\xi }^{mean}$, among others.

(6) The study was conducted on a laptop equipped with an NVIDIA 4070 GPU. We evaluated the runtime efficiency of the proposed AMSFF-Net model along with 11 other typical models, with the specific values shown in Table 3. As seen in Table 3, the runtime efficiency of our proposed model is higher than that of the three state-of-the-art models.

(7) Ablation Experiments: In this study, key components were removed from AMSFF-Net to test the performance of each component on the Focused-Defect-Detection-Tiles dataset. In this study, we employed $S_{\alpha }$, M, $E_{\xi }^{max}$, and $F_{\beta }^{w}$ from Table 2 as quantitative metrics to evaluate AMSFF-Net and its associated architectures.

Each technique adopted in the model was systematically removed one by one, resulting in the benchmark configuration B as shown in Table 4. The baseline results are presented in the first row of the table. Through comparative analysis, this paper specifically focuses on examining how different configurations affect model performance. The findings indicate that when random rotation or aspect ratio adjustment is applied individually, only a marginal improvement is observed, suggesting their limited contribution to overall performance when used alone. Nevertheless, documenting these findings has important implications for understanding the role and interaction of each technology within a given context. Particularly in real-world application scenarios, even slight performance enhancements can have significant impacts on task outcomes. Therefore, although this part of the experiment demonstrates limited performance gains, it plays a critical role in revealing both the relative importance and potential complementary roles of individual components within the model. When only the AMSFF module is incorporated, a significant improvement in model performance is observed compared to baseline B, achieving a 1.12% increase on $F_{\beta }^{w}$. When combining aspect ratio adaptation, random rotation augmentation, and AMSFF, the proposed AMSFF-Net enhances $S_{\alpha }$ by 0.56%, M remains unchanged, while $E_{\xi }^{max}$ and $F_{\beta }^{w}$ improve by 0.76% and 1.67%, respectively. The results verify the effectiveness of the method proposed in this study.

## 5. Conclusions

This study proposes a novel AMSFF-Net specifically to solve the SOD problem in surface defect images in industrial scenarios. By fusing adaptive weight fusion, global feature adaptive fusion, and differential feature adaptive fusion, AMSFF-Net effectively fuses information at different scales, thereby enhancing the accuracy of SOD. Additionally, the introduction of SA further enhances the fusion effect of feature maps. In addition, the recognition ability of the model for defects of different shapes, sizes, and angles is improved by preprocessing techniques such as adjusting the aspect ratio and random rotation. After rigorous screening and elimination of the ‘free’ and ‘uneven’ defect categories from the Magnetic-Tile-Defect dataset, it is renamed as the Focused-Defect-Detection-Tiles dataset. Through multiple experiments, the proposed method slightly underperforms PANet on $E_{\xi }^{max}$, but outperforms existing advanced techniques across all other metrics.

In future work, we will focus on adding additional defect types to expand the Magnetic-Tile-Defect dataset to address the current class imbalance issue. Moreover, we will explore advanced multi-scale feature fusion techniques to solve the problem where defects with significant size variations or very small defects are difficult to detect at certain scales.

## Figures and Tables

**Table 1:** Parameter settings of the neural network.

Parameter	Values	Parameter	Values
Training Cycles	150	Learning Rate Decay Type	Poly
Learning Rate	0.0015	Learning Rate Decay Rate	0.9
Weight Decay	$5\times 10^{-4}$	Batch Size	8
Print Frequency	50	Input Size	256 × 256
Momentum	0.9		

**Table 2:** The quantitative performance of nine state-of-the-art methods on the Focused-Defect-Detection-Tiles dataset was compared; ↑/↓ indicates that higher/lower scores are preferred.

Method	Backbone	Type	Module	$S_{\alpha }↑$	M↓	$E_{\varepsilon }^{adp}↑$	$E_{\varepsilon }^{mean}↑$	$E_{\varepsilon }^{max}↑$	$F_{\beta }^{adp}↑$	$F_{\beta }^{mean}↑$	$F_{\beta }^{max}↑$	$F_{\beta }^{\omega }↑$
FPN [35]	Res101	CN.	59.73	0.8550	0.0085	0.8825	0.9302	0.9485	0.6927	0.7585	0.7811	0.7594
RetinaFPN [35]	Res101	CN.	21.35	0.8671	0.0099	0.9285	0.9522	0.9627	0.7647	0.8332	0.8492	0.8396
EMANet [36]	Res101	CS.	22.41	0.8659	0.0054	0.9510	0.9689	0.9782	0.7784	0.8176	0.8336	0.8315
EDRNet [31]	Res34	CN.	39.15	0.8806	0.0060	0.9598	0.9571	0.9623	0.8218	0.8374	0.8503	0.8510
PANet [37]	Res50	CS.	36.18	0.8801	0.0054	0.9287	0.9696	0.9826	0.7464	0.8094	0.8341	0.8249
U2Net [38]	Residual U-Block (RSU)	CS.	1.13	0.8800	0.0063	0.9587	0.9689	0.9763	0.8016	0.8391	0.8609	0.8552
DACNet [32]	Res34	CR.	98.39	0.8902	0.0059	0.9668	0.9641	0.9741	0.8260	0.8486	0.8615	0.8582
A3Net_Res2Net50 [17]	Res2_50	CS.	31.08	0.8983	0.0049	0.9536	0.9693	0.9780	0.8019	0.8386	0.8632	0.8598
A3Net_VGG16 [17]	VGG16	CS.	17.32	0.8981	0.0048	0.9661	0.9670	0.9717	0.8266	0.8447	0.8675	0.8633
CSEPNET [34]	VGG16	CS.	19.36	0.8896	0.0048	0.9747	0.9704	0.9766	0.8429	0.8563	0.8677	0.8727
EMFINet [33]	Res34	CR.	99.13	0.9007	0.0050	0.9723	0.9691	0.9743	0.8425	0.8561	0.8688	0.8725
Ours	VGG16	CS.	17.39	0.9038	0.0048	0.9762	0.9733	0.9793	0.8445	0.8611	0.8782	0.8800

CN: CNN-based NSI-SOD method; CR: CNN-based RSI-SOD method; CS: CNN-based SDI-SOD method.

**Table 3:** Comparison of runtime efficiency between AMSFF-Net and 11 typical models.

Method	FPN	RetinaFPN	EMANet	EDRNet	PANet	U2Net	DACNet	A3Net_Res2Net50	A3Net_VGG16	CSEPNET	EMFINet	Ours
Params (M)	59.73	21.35	22.41	39.15	36.18	1.13	98.39	31.08	17.32	19.36	99.13	17.39
Speed (FPS)	21.75	14.61	23.85	5.78	22.89	12.78	3.80	22.92	15.40	12.66	3.58	15.57

**Table 4:** Ablation Study of Module Contributions in AMSFF-Net.

Settings	$S_{\alpha }$↑	M↓	$E_{\varepsilon }^{max}$↑	$F_{\beta }^{\omega }$↑
B	0.8982	0.0048	0.9717	0.8633
w/o aspect ratio	0.9004	0.0048	0.9749	0.8755
w/o random rotation	0.8981	0.0045	0.9790	0.8795
w/o AMSFF	0.8934	0.0048	0.9757	0.8662
w AMSFF	0.8995	0.0048	0.9780	0.8745
Ours	0.9038	0.0048	0.9793	0.8800

## Data Availability

The data for this article are not publicly available, but will be made available upon request.
